# SPIO-enhanced MRI for sentinel lymph node mapping in oral cancer: a prospective feasibility study

**DOI:** 10.1186/s41747-025-00636-4

**Published:** 2025-11-15

**Authors:** Gijs T. N. Heldens, Daphne A. J. J. Driessen, Tim Dijkema, Anne I. J. Arens, Patrik Zámecnik, Sjoert A. H. Pegge, Willem L. J. Weijs, Adriana C. H. van Engen-van Grunsven, Robert P. Takes, Johannes H. A. M. Kaanders, Tom W. J. Scheenen

**Affiliations:** 1 Department of Otorhinolaryngology and Head and Neck Surgery, Radboud University Medical Center, Nijmegen, The Netherlands; 2 Department of Radiation Oncology, Radboud University Medical Center, Nijmegen, The Netherlands; 3 Department of Medical Imaging, Radboud University Medical Center, Nijmegen, The Netherlands; 4 Department of Oral- and Maxillofacial Surgery and Head and Neck Surgery, Radboud University Medical Center, Nijmegen, The Netherlands; 5 Department of Pathology, Radboud University Medical Center, Nijmegen, The Netherlands

**Keywords:** Lymphatic metastasis, Magnetic iron oxide nanoparticles, Magnetic resonance imaging, Single photon emission computed tomography/computed tomography, Tongue neoplasms

## Abstract

**Background:**

Patients with early-stage node-negative oral cancer undergo a sentinel lymph node biopsy (SLNB) or elective neck dissection under general anesthesia. A noninvasive imaging alternative would be of great interest. Superparamagnetic iron oxide (SPIO)-enhanced magnetic resonance imaging (MRI) can visualize draining lymph nodes and potentially metastases. We investigated the optimal combination of SPIO injection and T2*-weighted MRI settings to identify the sentinel lymph nodes (SLNs), the lymphatic drainage pattern, and possibly to detect metastatic SLNs.

**Materials and methods:**

SPIO nanoparticles were injected submucosally around the primary tongue tumor in ten patients after routine SLNB imaging with indocyanine green-[^99m^Tc]Tc-nanocolloid, and MRI was performed 1 h after injection. SPIO dose was adjusted for every two patients based on the imaging quality. Drainage patterns were compared between single-photon emission computed tomography (SPECT)/computed tomography (CT) and MRI. MRI appearance of SLNs was compared to histopathology of resected nodes.

**Results:**

One mg of iron was deemed a suitable dose after two dose alterations. All 25 SLNs observed on SPECT/CT in eight patients were also identified on MRI. Including higher echelons, 55 lymph nodes were seen on SPECT/CT, while 107 lymph nodes were seen on MRI. Eighteen lymph nodes showed a mixture of partial MRI signal attenuation and retention, which, when compared to histopathology, made discrimination between metastatic and nonmetastatic lymph nodes solely based on MRI impossible.

**Conclusion:**

SPIO-enhanced T2*-weighted MRI is suitable for mapping SLNs and lymphatic drainage pattern, showing significantly more lymph nodes compared to SPECT/CT. Discriminating metastatic from nonmetastatic nodes does not seem feasible after SPIO injection.

**Trial registration:**

Clinicaltrials.gov NCT04803331. Registered 4 March 2021; https://clinicaltrials.gov/study/NCT04803331.

**Relevance statement:**

SPIO-enhanced MRI seems noninferior to the current method of SLN detection with technetium, with better anatomical detail than SPECT/CT: if proven comparable, SPIO-enhanced MRI could be considered a nonradioactive alternative with higher spatial resolution to define lymphatic drainage of tumors.

**Key Points:**

- The use of superparamagnetic iron oxide (SPIO)-enhanced MRI in head and neck cancer is underassessed.
- SPIO-enhanced MRI detects nodal drainage patterns comparably to SPECT/CT.
- SPIO-enhanced MRI does not discriminate lymph node metastases from false positives.

**Graphical Abstract:**

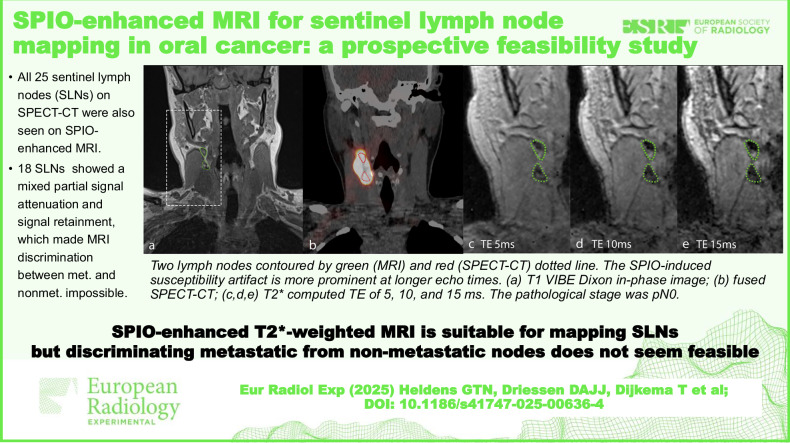

## Background

The sentinel lymph node biopsy (SLNB) has become the standard of care to stage the neck in patients with early-stage oral cavity squamous cell carcinoma and a clinically negative neck (cN0) over the past 10 to 15 years [1]. In this procedure, a radioactive tracer is peritumorally injected and drains into cervical lymph nodes, mimicking the potential route of lymph drainage of tumor cells. Using planar lymphoscintigraphy and single-photon emission computed tomography (SPECT)/computed tomography (CT), a nuclear medicine physician annotates the sentinel lymph nodes (SLNs). During the surgery of the primary tumor, the SLNs are reidentified using a gamma probe and excised. Subsequently, a pathologist analyzes the SLNs using step-serial sectioning. The SLNB has significant advantages compared to alternative, more extensive surgical strategies for the cN0 neck [2]. However, it still comprises a surgical intervention and requires a nuclear medicine department to perform a procedure that takes a long time with cumbersome logistics, scheduling imaging and surgery within a suitable period. A noninvasive diagnostic imaging procedure to stage the neck without the need for a radioactive tracer would be of great clinical interest.

Superparamagnetic iron oxide (SPIO) nanoparticles have been investigated as an alternative nonradioactive tracer for SLNB [3]. After peritumoral injection, these particles also drain via the lymphatic system into SLNs [4–6], where they act as a contrast agent to identify sentinel nodes using iron-sensitive MRI [7–10]. Their superparamagnetic iron oxide core generates a local magnetic field inhomogeneity that causes strong MR signal attenuation in T2* weighted MRI sequences. Combined with the high spatial resolution of MRI (compared to other functional imaging methods like PET or SPECT), this makes SPIO nanoparticles a highly potent MRI contrast agent to visualize SLNs [11]. There is some evidence that SPIO particles, similar to ultrasmall SPIOs (USPIOs), could be differentially taken up in metastatic and nonmetastatic (parts of) lymph nodes, which could help in discriminating metastatic from nonmetastatic SLNs [12]. Contrary to locally injected SPIOs, USPIOs (with a size of 17–20 nm *versus* 59 nm of SPIO) are administered intravenously [13, 14]. These particles are phagocytosed by cells of the immune system over 24–36 h after injection, where they locally accumulate in healthy lymph nodes, promoting strong signal attenuation. Metastases do not accumulate these particles and retain signal, making USPIO-enhanced MRI suitable for detecting metastases [15, 16]. If SPIO accumulates inside lymph nodes in a similar way within the time frame of 30 to 60 min from injection to detection, this could offer an opportunity not only to identify the SLN, but also to radiologically assess the presence of metastases within the SLN [12, 17–19]. This assessment is based on the presence or absence of partial signal intensity in the lymph node.

While many publications have explored the use of SPIO as an interstitial MRI contrast agent in different cancers, only a few have investigated its feasibility for SLN mapping in oral cancer cohorts using SPIO-enhanced MRI [10, 20, 21].

In previous reports, the MRI pulse sequences and field strengths varied, and the dose of SPIO varied from 0.1 to 0.4 mL [10, 20, 21]. Therefore, this study aimed to evaluate the optimal dose and pulse sequence for SPIO-enhanced MRI at 3 T in oral cancer patients to detect the SLNs using [^99m^Tc]Tc-nanocolloid injection and SPECT/CT as the reference standard. With histopathology and high-resolution *ex vivo* MRI of the resected SLNs, the feasibility of detecting metastases in the SLNs was investigated as well.

## Materials and methods

### Patients

This research was approved by the institutional review board of the Radboud University Medical Center (CMO Arnhem-Nijmegen), and written informed consent was obtained from all patients. The study was registered on 4 March 2021 in clinicaltrials.gov (NCT04803331).

Between July 2021 and April 2023, we included ten patients with T1 or T2 oral cavity squamous cell carcinoma of the lateral tongue, classified as cN0, planned to undergo primary tumor resection and SLNB. cN0 was determined by routine clinical assessment, including physical examination, ultrasound with fine-needle aspiration in suspicious lymph nodes, and CT or MRI on indication. Exclusion criteria were previous surgery or radiotherapy to the head or neck, extensive dental restorations visualized on panoramic x-ray, pregnancy, and a contraindication for MRI or SPIO.

### Routine SLN imaging

Routine SLN imaging was based on the European Association of Nuclear Medicine consensus paper [22]. One day before surgery, four 0.15 mL (0.1–0.2 mL) aliquots, each containing 20 MBq of indocyanine green (ICG)-[^99m^Tc]Tc-nanocolloid, were injected at four locations around the primary tumor using a 27 G needle after locally administered xylocaine spray. These locations were marked using nonmetallic medical ink to ensure later identical SPIO injection placement. Static planar and SPECT/CT imaging were subsequently performed. SPECT acquisition parameters included a matrix of 256 × 256, noncircular type, step-and-shoot rotation, and 180° clockwise rotation with 20 views per detector. Low-dose CT acquisition parameters included 40 mAs, 110 kVp, 5.0-mm slices (acquisition 2 × 2.5 mm), and 1.2 pitch. Transversal, coronal, and sagittal SPECT and low-dose CT images were fused.

### SPIO-enhanced SLN MRI

After SPECT/CT, a SPIO (Magtrace^®^, Endomagnetics Ltd) dilution was injected with a 27 G needle in the four marked locations after locally administered xylocaine spray. Magtrace^®^ consists of an aqueous carboxydextran-coated superparamagnetic iron oxide suspension formulated with 0.3% (weight/volume) sodium chloride [23].

In line with previous work with a similar SPIO particle, a total amount of 0.012 mL SPIO containing 0.33 mg of iron was administered to the first two patients in our study (SPIO diluted in 0.9% NaCl to obtain a total volume of 0.4 mL, distributed over 4 injections) [12]. This dose was adjusted for the next patients (see below). Patients were asked to report pain on a numeric pain rating scale from 0 to 10 after the injection of both agents. MRI examinations were performed approximately 1 h after SPIO injection on a 3-T system (Magnetom Prisma-Fit, Siemens Healthineers, Erlangen, Germany) with a 24-channel head-neck coil and an additional 8-element flex coil on the upper chest. The protocol consisted of three-dimensional T1-weighted Dixon volume interpolated breath-hold examination (VIBE) images and three-dimensional multigradient-echo (MGRE) T2*-weighted images. Dixon imaging provided water, lipid, in-phase, and opposed-phase image sets. An isotropic resolution of 0.8 × 0.8 × 0.8 mm^3^ was used. Dedicated custom-made software (in MATLAB) was used to fit a mono-exponential decay curve to the T2* signal attenuation and to compute different echo time images from the MGRE pulse sequence [24]. Details of the pulse sequences are shown in Table 1. While the T1-weighted pulse sequence served as an anatomical guide commonly used in clinical practice, the radiologist used the T2*-weighted sequence to detect all nodes containing SPIO, using the different computed echo times with the blooming effect to gain information on the size of the artifact.

**Table 1:** *In vivo* and *ex vivo* MRI parameters

	*In vivo*		*Ex vivo*	
	T1 VIBE Dixon	T2* MGRE	Water excitation	Lipid excitation
Resolution (mm^3^)	0.8 × 0.8 × 0.8	0.8 × 0.8 × 0.8	0.0625 × 0.0625 × 0.0625	0.0625 × 0.0625 × 0.0625
Acquisition mode	3D coronal	3D coronal	3D coronal	3D coronal
Field of view (mm)	260 × 260	260 × 260	25 × 16 × 5	25 × 16 × 5
Echo time (ms)	2.57, 3.8	2.5–27.1^a^	2.3–17.3^b^	2.8
Repetition time (ms)	6.02	31	60	30
Bandwidth (Hz/pixel)	300	360	520	520
Acquisition time (min)	04:53	11:50	Variable	Variable
Flip angle (°)	10	10	10	10

*MGRE* Multigradient-echo, *MRI* Magnetic resonance imaging, *VIBE* Volume interpolated gradient-echo

^a^ 6 in-phase echoes

^b^ 8 echoes, equally distributed

### Dose optimization

An experienced radiologist qualitatively evaluated T1- and T2*-weighted MGRE images after every two patients to determine the next step in dose optimization. If there was little signal attenuation due to SPIO presence, the dose was elevated for subsequent patients. The dose was lowered if there was too much signal attenuation to delineate the lymph node and recognize surrounding structures. Once a specific dose provided satisfactory images, subsequent patients would receive the same dose.

### Diagnostic performance evaluation

Using T1- and T2*-weighted MGRE images, the radiologist assessed which (sentinel) lymph nodes contained a residual signal-intense area. These areas were thought to be signal-intense because a possible metastasis prevents the presence of macrophages responsible for SPIO uptake, which gives signal attenuation through SPIO concentration in healthy nodes. After resection, the status of the radiologically suspicious nodes showing a signal-intense area was correlated with *ex vivo* high-resolution MRI and histopathological findings of these nodes.

### Surgery

Following clinical routine for these patients, after imaging of the SLNs with the SPECT/CT, surgery was performed within 24 h, and the SLNs were resected using a gamma probe intraoperatively. The SLNB was based on the consensus surgical guidelines [1].

### *Ex vivo* MRI

Excised SLNs were sectioned in 2.0–3.0-mm thick sections, placed in a tissue cassette, and fixated in 10% buffered formalin for at least 12 h. After fixation, an *ex vivo* MRI of the cassette was performed on a preclinical 11.7-T system (Bruker BioSpec; BioSpin MRI GmbH, Ettlingen, Germany) with a cryogenic surface coil (Bruker CryoProbe™; BioSpin MRI GmbH) for iron-sensitive MRI with maximum sensitivity (details in Table 1).

### Histopathology

After *ex vivo* MRI, histopathological analysis of the SLNs was performed, including hematoxylin and eosin staining, immunohistochemistry with pan-cytokeratin (CK-PAN), and iron staining (Perls’ Prussian blue stain). The *ex vivo* images of metastatic lymph nodes were compared to the pathology slides to identify whether the MRI signal intensity pattern corresponded with the staining patterns. The *ex vivo* image orientation was matched to the histopathology slice orientation using anatomical landmarks and the surrounding lipid tissue. Nodes on *ex vivo* MRI could be matched with *in vivo* MRI using morphological and attenuation-pattern correspondence.

### SPECT/CT and MRI *in vivo* correlation

An experienced nuclear medicine physician assessed the SPECT/CT images. All lymph nodes containing technetium signals were annotated, including SLNs and higher echelon nodes. On SPIO-enhanced MRI, the radiologist annotated all lymph nodes showing signal attenuation. Both imaging specialists were blinded to each other’s findings, ensuring unbiased interpretation. Based on anatomical location and morphology, concordance was assessed between the detected SLNs and higher echelon nodes on SPECT/CT and MRI. After unblinding, lymph nodes only annotated on SPECT/CT were identified on MRI and assessed for a second time by the radiologist for the presence of SPIO.

### Statistical analysis

Descriptive statistics were performed using IBM SPSS Statistics version 29. The Wilcoxon Signed-Rank test and the Spearman’s ρ test were used for ordinal data of paired samples and non-normally distributed data, respectively. Concordance was calculated as the percentage with 95% confidence intervals of (sentinel) lymph nodes annotated on SPECT/CT that were also annotated on SPIO-enhanced MRI. A *p*-value lower than 0.05 was considered statistically significant.

## Results

### Patient characteristics

Ten patients were enrolled in this study: 7 males and 3 females. The median age was 64 years, ranging from 39 to 73. Table 2 shows detailed baseline characteristics.

**Table 2:** Patient characteristics

Patient	Age (years)	Sex	Side	pT	pN	Magtrace dose (mL; mg iron)	SPIO-to-MRI interval (min)
1	71	Male	Right	2	2b	0.012; 0.34	63
2	55	Male	Left	2	1	0.012; 0.34	58
3	71	Male	Right	2	0	0.12; 3.36	57
4	69	Male	Right	1	0	0.12; 3.36	53
5	66	Female	Left	2	0	0.036; 1.01	28
6	52	Male	Right	1	0	0.036; 1.01	50
7	73	Female	Left	2	0	0.036; 1.01	50
8	62	Male	Right	1	0	0.036; 1.01	28
9	51	Male	Left	2	2b	0.036; 1.01	64
10	39	Female	Right	1	0	0.036; 1.01	60

Tumor stage was classified using the eighth edition TNM Classification of Malignant Tumors of the Union for International Cancer Control

*MRI* Magnetic resonance imaging, *N* Node, *p* Pathologic, *SPIO* Small particle iron oxide, *T* Tumor

#### Tolerability

Pain scores on a numeric pain rating scale from 0 to 10 were higher after SPIO injection compared to ICG-[^99m^Tc]Tc-nanocolloid injection (median 6.00 (range 4–10] *versus* 3.50 (2–7] (Wilcoxon signed-rank, *p* = 0.007)). No significant differences in pain scores were noted between different doses (Spearman’s ρ, *p* ≥ 0.697). No adverse events occurred, such as swelling on the primary injection site or clinically relevant hematoma [20].

### SPIO dose finding and MRI signal attenuation

In the first two patients, the first dose resulted in marginal visibility of SPIO-induced signal attenuation on T1- and T2*-weighted imaging. The second pair of patients received 0.12 mL of SPIO, leading to excessive blooming artifacts covering the entire lymph node and signal attenuation occasionally overlapping with nearby other lymph nodes. A dose of 0.036 mL of SPIO was chosen for patients 5 and 6, obtaining satisfactory imaging results. The boundaries of the lymph nodes with a near absence of signal were clearly visible in the T1-weighted VIBE Dixon acquisition and at the first echo time of the MGRE sequence, while the size of the susceptibility artifact increased at later echo times. This dose was subsequently also administered to the last four patients.

### SPECT/CT and MRI *in vivo* correlation

As MRI signal attenuation was marginal in the first two patients, these patients were excluded from this analysis. Therefore, these results concern only patients 3 to 10. A patient flow chart is provided in Appendix 1.

On SPECT/CT, 2 to 5 SLNs per patient were designated for excision, totaling 25 SLNs in 8 patients. Of these 25 SLNs, 24 (96%, 95% confidence interval: 79.7–99.9%) were detected on initial MRI. Four to 12 higher echelon lymph nodes per patient were seen on SPECT/CT for a total of 30 lymph nodes, of which 23 (77%, 57.7–90.1%) were identified on MRI on initial assessment.

**Fig. 1:**
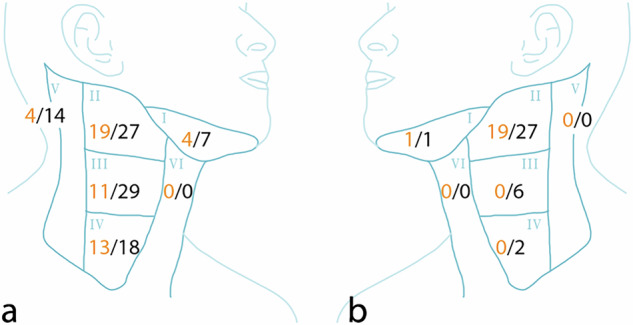
Diagram of lymph node distribution of 8 patients on SPECT/CT (orange numbers) and SPIO-enhanced MRI (black numbers) after unblinding and secondary radiological assessment. **a** Ipsilateral; **b** contralateral

**Fig. 2:**
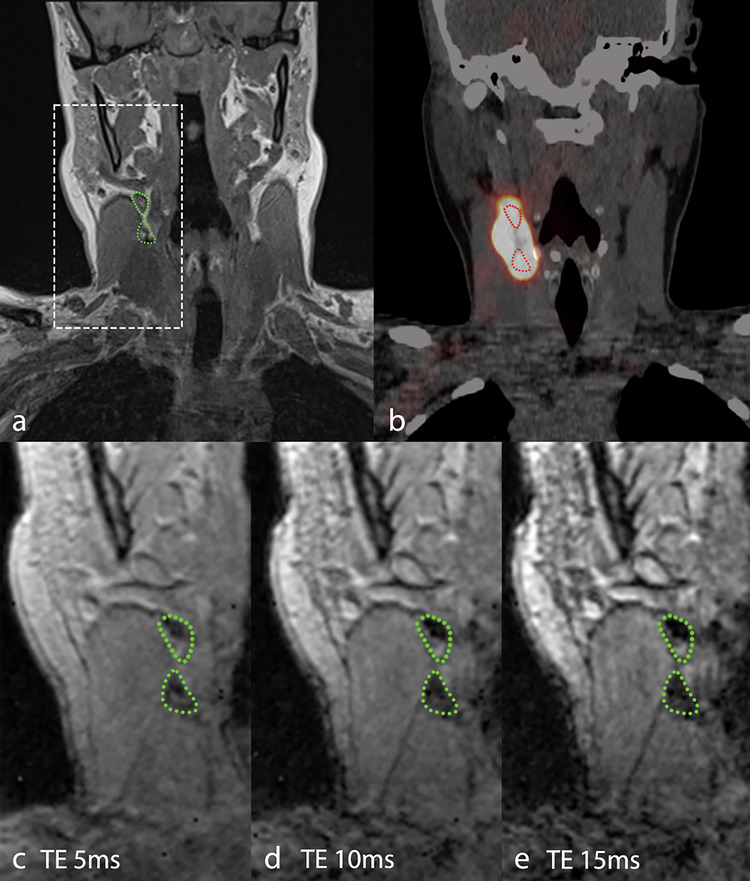
Coronal images of patient #10 with a right-sided lateralized tongue tumor after injection of 0.036 mL of SPIO. **a** T1-weighted VIBE Dixon in-phase image; **b** fused SPECT/CT; T2*-weighted images with computed TE of 5 ms (**c**), 10 ms (**d**), and 15 ms (**e**). The pathological nodal stage was pN0. Two lymph nodes are indicated by the green (MRI) and red (SPECT/CT) dotted line; the SPIO-induced susceptibility artifact is more prominent at longer echo times. MRI, Magnetic resonance imaging; SPECT/CT, Single-photon emission computed tomography/computed tomography; SPIO, Small particle iron oxide; TE, Echo time; VIBE, Volume interpolated gradient-echo

Comparing the two techniques with retrospective unblinded assessment, 25/25 (100%, 86.3–100.0%) of the SLNs on SPECT/CT showed MR signal attenuation. Of 30 higher echelon nodes, 28 (93%, 77.9–99.2%) were seen on MRI. In total, 53 of the 55 nodes (SLNs plus higher echelon nodes) identified on SPECT/CT were also identified on MRI. Conversely, many more nodes were identified on MRI: 107 nodes in total. Level-based drainage is shown in Table 3 and Fig. 1. One SPECT/CT-identified lymph node did not show SPIO uptake, and the evaluation of another lymph node was hindered by motion artifacts and distortion due to local magnetic field inhomogeneities that occur at interfaces between tissues. Both nodes were assigned as higher echelon lymph nodes on SPECT/CT and were of no significant clinical relevance. The T2*-weighted MGRE sequence proved helpful in identifying SPIO-containing nodes, especially in the nodes showing little attenuation, as shown in Fig. 2.

**Table 3:** Level-based lymphatic drainage

Level	SPECT/CT (%)	SPECT/CT SLN (%)	SPIO-MRI (%)
I	5 (9.1)	3 (12)	8 (7.5)
II	22 (40)	17 (68)	30 (28)
III	11 (20)	5 (20)	35 (32.7)
IV	13 (23.6)	0	20 (18.7)
V	4 (7.3)	0	14 (13.1)
Total	55	25	107

*SPECT/CT* Single-photon emission computed tomography/computed tomography, *MRI* Magnetic resonance imaging, *SLN* Sentinel lymph node, *SPIO* Small particle iron oxide

**Fig. 3:**
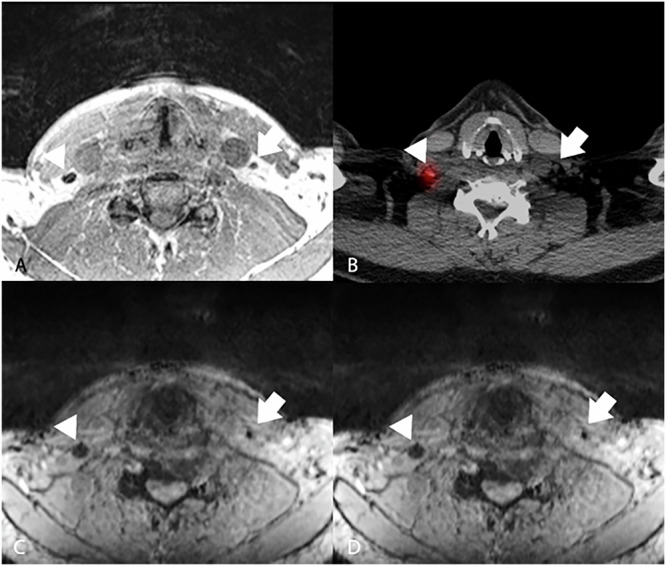
Transversal images of patient #6 with a right-sided lateralized tongue tumor after injection of 0.036 mL of SPIO. **A** T1-weighted VIBE Dixon, in-phase image; **B** fused SPECT/CT; T2*-weighted images computed TE of 5 ms (**C**) and 10 ms (**D**). Arrowheads indicate ipsilateral drainage of both ICG-[^99m^Tc]Tc-nanocolloid and SPIO on SPECT/CT and MRI. Arrows indicate contralateral drainage of SPIO, whereas no drainage of ICG-[^99m^Tc]Tc-nanocolloid is seen on the SPECT/CT. ICG, Indocyanine green; MRI, Magnetic resonance imaging; SPECT/CT, Single-photon emission computed tomography/computed tomography; SPIO, Small particle iron oxide; TE, Echo time; VIBE, Volume interpolated gradient-echo

In two patients with the highest administered SPIO dose (0.12 mL), the total number of detected lymph nodes on MRI was highest (18 and 28 nodes *versus* median 11 (range 4–15) in 6 patients with lower dose, Spearman’s ρ, *p* = 0.050). Two patients showed bilateral drainage on MRI and SPECT/CT. One patient showed bilateral drainage on SPECT/CT and ipsilateral drainage on MRI. One patient showed contralateral drainage on MRI in several nodes in level III, while there was no contralateral drainage of the radioactive tracer (Fig. 3). As these nodes were not seen on SPECT/CT, they were not resected. After 18 months of follow-up, this patient showed no sign of regional recurrence.

Of the six patients injected with the optimal SPIO dose of 0.036 mL, only two SLNs in one patient contained metastases. As these SLNs were excised based on the conventional SLN procedure with SPECT/CT and gamma probe and not based on SPIO-enhanced MRI, a direct node-to-node comparison between *in vivo* MRI with histopathology was inconclusive. Nine histopathological nonmetastatic SLNs showed a mixture of partial MRI signal attenuation and signal retention within the node on *in vivo* MR imaging. Another 9 higher echelon nodes showed the same partial signal retention.

### MRI *ex vivo*/histopathology correlation

**Fig. 4:**
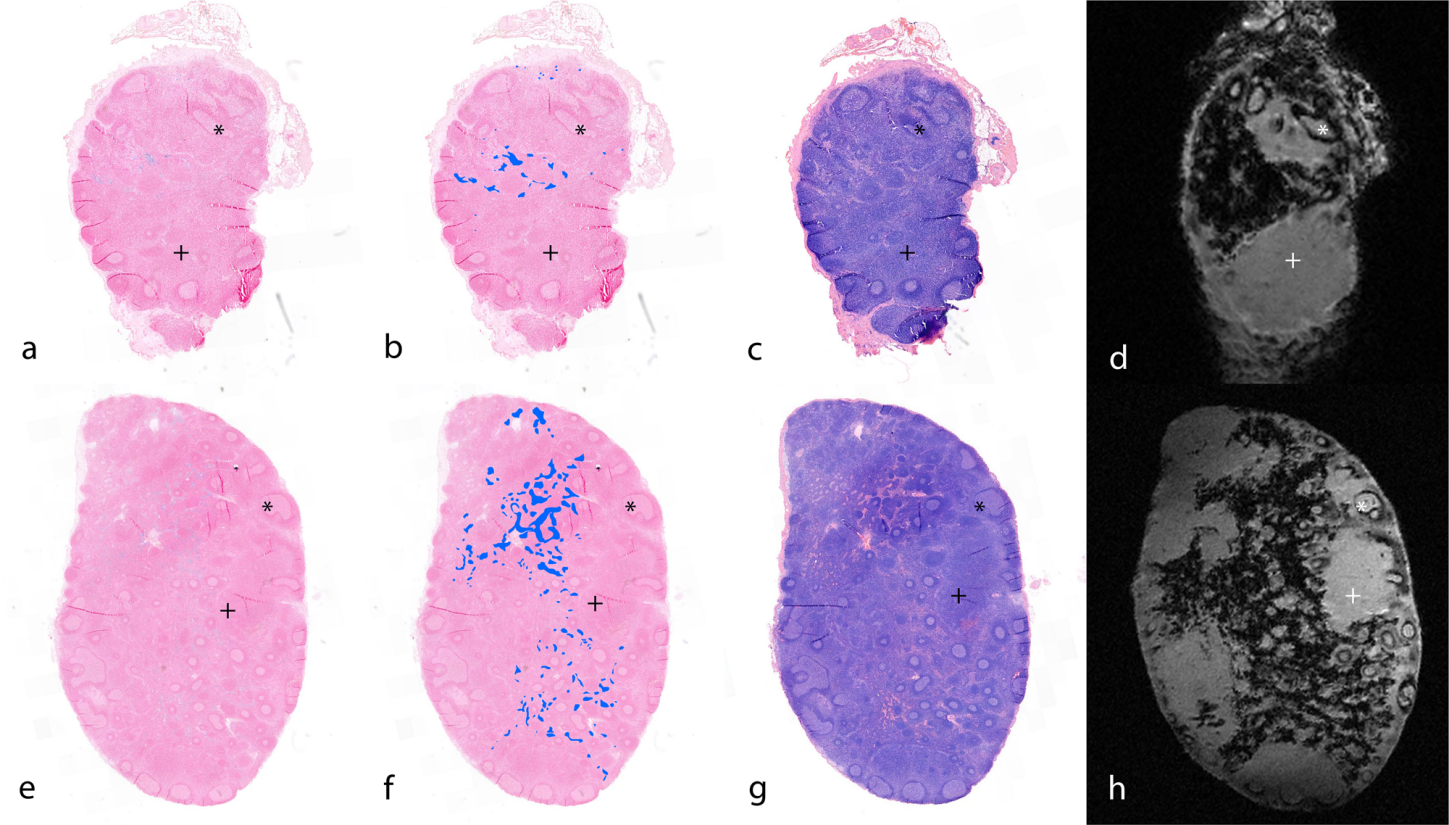
Matched histopathology and *ex vivo* MRI of two sentinel lymph nodes. Perls’ Prussian blue histopathological staining (**a**, **e**), Perl’s image with stained areas marked as blue (**b**, **f**), hematoxylin and eosin stain (**c**, **g**), and *ex vivo* MRI (**d**, **h**) of two sentinel lymph nodes. The presence of SPIO corresponds to histopathology and MRI, respectively seen as the Prussian blue areas and the areas of signal attenuation. Note the distinct absence of SPIO in areas without sinuses (+) and follicles (*). MRI, Magnetic resonance imaging; SPIO, Small particle iron oxide

All SLNs annotated on SPIO-enhanced MRI showed iron depositions after staining with Perls’ Prussian blue (iron staining). The susceptibility artifact on *ex vivo* T2*-weighted imaging corresponded with the Perls’ staining on histopathology (Fig. 4). Histopathology indicated that the artifact was primarily localized within lymph node sinuses containing macrophages, while follicles and areas without sinuses showed no SPIO presence on histopathology or MRI.

## Discussion

While SPIO is a promising tracer and MRI contrast agent, its clinical application remains underexplored. This study aimed to identify optimal settings for obtaining detailed MRI to assess lymphatic drainage patterns in the head and neck and explore the potential for detecting metastases in SLNs.

The first SPIO dose of 0.012 mL in this study was chosen based on a study on SLNs in breast cancer [12]. This dose proved to be inadequate for our aim, possibly caused by differences between the primary injection sites (submucosal in the tongue and subdermal in the breast) and lymphatic drainage to the axilla and neck. Using the second SPIO dose of 0.12 mL, the susceptibility artifact precluded adequate assessment of adjacent lymph nodes. After the second adjustment, an infusion of 0.036 mL SPIO was established as optimal for effectively identifying SLNs using SPIO-enhanced MRI. All SLNs seen on SPECT/CT were marked on MRI with this dose. The T2*-weighted MGRE pulse sequence proved to be instrumental in identifying SPIO-containing nodes, as it allowed visualization of the amplification of the attenuation artifact at longer echo times, particularly useful in nodes exhibiting minimal attenuation. Pain scores when injecting SPIO were significantly higher than ICG-[^99m^Tc]Tc-nanocolloid injections. One previous notion about higher pain scores after SPIO injections can be found in the literature, reporting a lower pain score in one patient after administration of xylocaine spray [21]. Some other studies report equal pain scores compared to [^99m^Tc]Tc-nanocolloid or local anesthetics before administration [12, 25, 26].

A surplus of 52 nodes was detected on MRI compared to SPECT/CT, indicating that the SPIO particles penetrated further into the lymphatic system of the neck than ICG-[^99m^Tc]Tc-nanocolloid in a shorter time interval. While this does not improve sensitivity for detecting metastatic nodes, it could better depict the lymphatic drainage compared to radioactive tracers. This can be useful in tailoring target areas for elective radiotherapy of the neck in head and neck cancers, as proposed in the literature [27]. Coating, charge, and nanoparticle size (4–100 nm (95% of the particles are less than 80 nm in size) for [^99m^Tc]Tc-nanocolloid and 45–65 nm for SPIO) are crucial factors influencing lymph drainage and could explain the larger drainage pattern seen with MRI [23, 28]. Comparing SPECT/CT to MRI, six lymph nodes with a weak SPECT/CT signal were only seen after unblinding, presumably due to a likewise small amount of SPIO present. All but two higher echelon nodes were concordant with SPECT/CT, one of which was a contralateral node. Artifacts hindered the evaluation of the other lymph node on MRI.

In this study, MRI was performed approximately 1 h after SPIO injection. Previous studies focused on detecting SLNs suggested a 10-min interval for the detection of sentinel nodes [10, 20]. Mizokami et al [20] used a 10-min and 24-h interval in 3 patients, accomplishing complete concordance (7/7 SLNs) at 10 min, and a surplus of over 31 nodes on MRI after 24 h. As we aimed to differentiate between metastatic and nonmetastatic sentinel lymph nodes, we chose a longer injection-imaging interval, enough for the SPIO to migrate to and accumulate in the lymph node parenchyma. The technique proposed relies on imaging at one time point, which requires the radiologist to interpret the images based on signal loss with echo time, drainage pattern and anatomical location rather than time of uptake. With 9 histopathologically nonmetastatic SLNs and another 9 higher echelon nodes with partial signal attenuation and partial signal retention, we can conclude that with our used strategy using SPIO-enhanced MRI to detect metastases in SLNs, a large number of false positives can be expected. As this would mean numerous needless therapeutic neck dissections, which are major surgical interventions, this approach is not clinically viable for the particular purpose of detecting metastases. These false positives could be caused by the injection-imaging interval or by benign processes such as follicular hyperplasia, like in USPIO imaging [29, 30]. A shorter interval between injection and MRI might result in a more restrictive and specific drainage pattern but could result in more signal-intense areas in the nodes. A longer interval could facilitate SPIO migration into all benign parts of lymph nodes, reducing the occurrence of false positives, but can also cause broader dispersion through the cervical lymphatics. Multiple imaging points would be preferable in the future, but would make this technique more labor-intensive. While USPIO-enhanced MRI 24–36 h after systemic intravenous administration seems suitable for detecting metastases in lymph nodes due to the particles’ lymphotropic properties, SPIO particles are bigger and seem more suitable to be used as a tracer of lymphatic drainage when administered interstitially [15, 31, 32]. Theoretically, [^99m^Tc]Tc-nanocolloid could negatively influence the drainage of the later injected SPIO because of accumulation in lymph nodes and lymph vessels. However, this is not expected, as SPIO shows a broader dispersion in this study, not a narrower dispersion.

This study is a relatively small feasibility study, with strong attributes such as the clinical reference standards of SPECT/CT and histopathology, confirming the ability of SPIO particles to identify SLNs and lymphatic drainage patterns. As the injection spots were marked, the variance in the injection locations was minimal. Also, equal volumes of SPIO solution and ICG-[^99m^Tc]Tc-nanocolloid were used. It is the first time SPIO presence is demonstrated in lymph nodes on 11.7-T *ex vivo* MRI with this level of detail, spatially matched with histopathology. As this technique eliminates the need for a nuclear medicine facility and the safety measures needed to handle radioactive tracers, lower procedural costs are expected compared to lymph node imaging with radioactive tracers. However, the potential of discriminating between metastatic and nonmetastatic SLNs with this procedure is limited, as matched *ex vivo* MRI and histopathology confirmed the absence of SPIO in certain nodal areas without the presence of metastases. The main limitations of this study are the small number of patients and metastases and the evaluation of only a single time interval between injection and *in vivo* MRI. While the retrospective unblinded node identification enabled a direct comparison of the drainage of the two contrast agents, this introduced a risk of recall bias. Lastly, MRI-pathology matching was limited due to the study’s design: SLNs on SPECT/CT were resected using a gamma probe. While the nodes on SPECT/CT and MRI were matched, resected nodes could not be matched for certain with the nodes on MRI.

Three-dimensional images at high spatial resolution were obtained using T2*-weighted MRI, visualizing SPIO particles in draining (sentinel) lymph nodes after interstitial injection. Lymphatic drainage mapping using SPECT/CT has been investigated for the de-escalation of elective radiotherapy of the neck, aiming to unilaterally radiate patients with no lymphatic flow to the node-negative contralateral neck in head and neck squamous cell carcinoma patients with a lateralized tumor [27]. If SPIO continues to prove itself comparable to ICG-[^99m^Tc]Tc-nanocolloid, it could be considered a nonradioactive alternative with high spatial resolution to define the lymphatic drainage of a tumor. Future research on its role in the management of the neck should therefore mainly be focused on its function as a lymphatic tracer. This future work could use multiple time points in a larger cohort of patients, containing more metastases. Artificial Intelligence-assisted interpretation of these SPIO-enhanced MRI images in the future should also be considered [33]. Overall, SPIO-enhanced MRI seems noninferior to the current method of SLN detection with technetium, with better anatomical detail than SPECT/CT. SPIO-enhanced MRI may not reliably detect metastases but shows potential as a high-resolution, nonradioactive alternative to SPECT/CT for lymphatic mapping.

## Supplementary information

ELECTRONIC SUPPLEMENTARY MATERIAL

## Data Availability

The datasets used and/or analyzed during the current study are available from the corresponding author upon reasonable request.

## Abbreviations

CT: Computed tomography
ICG: Indocyanine green
MGRE: Multigradient-echo
MRI: Magnetic resonance imaging
SLNB: Sentinel lymph node biopsy
SLNs: Sentinel lymph nodes
SPECT: Single-photon emission computed tomography
SPIO: Superparamagnetic iron oxide
USPIO: Ultrasmall superparamagnetic iron oxide
VIBE: Volume interpolated breath-hold examination
