# Supplementary material for: SPIO-enhanced MRI for sentinel lymph node mapping in oral cancer: a prospective feasibility study
Source: Eur Radiol Exp. 2025 Nov 15;9:113. doi: 10.1186/s41747-025-00636-4 (PMC12619859; doi:10.1186/s41747-025-00636-4)
Supplement: Supplementary file 1 — ELECTRONIC SUPPLEMENTARY MATERIAL [file 41747_2025_636_MOESM1_ESM.pdf]

SPIO-enhanced MRI for sentinel lymph node mapping in oral cancer: a prospective feasibility study

ELECTRONIC SUPPLEMENTARY MATERIAL

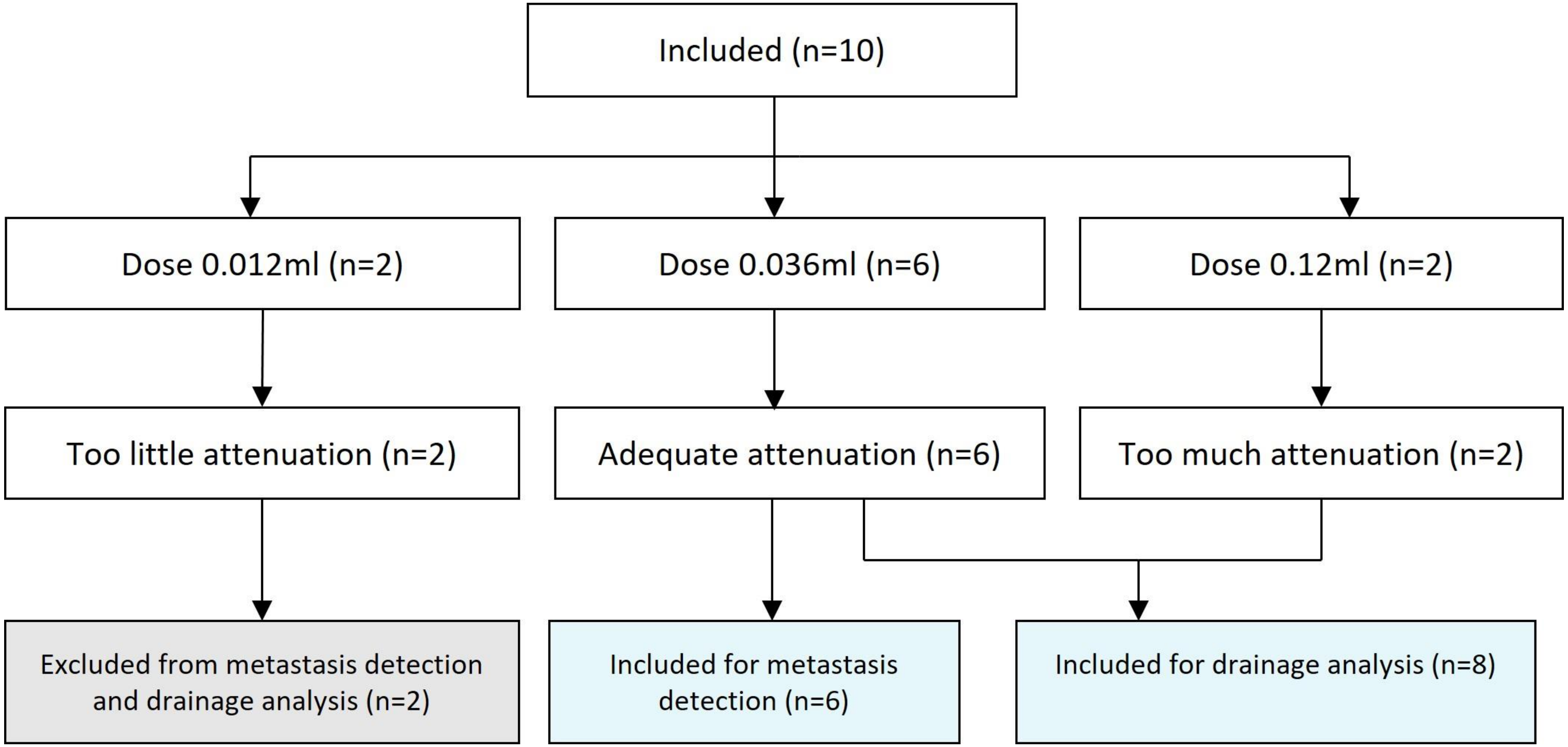

Appendix 1 *Patient flow diagram*
